# Dietary tributyrin, an HDAC inhibitor, promotes muscle growth through enhanced terminal differentiation of satellite cells

**DOI:** 10.14814/phy2.13706

**Published:** 2018-05-23

**Authors:** Robert L. Murray, Wei Zhang, Marie Iwaniuk, Ester Grilli, Chad H. Stahl

**Affiliations:** ^1^ Department of Animal and Avian Sciences University of Maryland‐College Park College Park Maryland; ^2^ Department of Veterinary Medical Sciences University of Bologna Ozzano Emilia Bologna Italy

**Keywords:** Butyrate, HDAC inhibitor, myogenesis, satellite cell, tributyrin

## Abstract

Muscle growth and repair rely on two main mechanisms – myonuclear accretion and subsequent protein accumulation. Altering the ability of muscle resident stem cells (satellite cells) to progress through their myogenic lineage can have a profound effect on lifetime muscle growth and repair. The use of the histone deacetylase (HDAC) inhibitor, butyrate, has had positive outcomes on the in vitro promotion of satellite cell myogenesis. In animal models, the use of butyrate has had promising results in treating myopathic conditions as well as improving growth efficiency, but the impact of dietary butyrate on satellite cells and muscle growth has not been elucidated. We investigated the impact of tributyrin, a butyrate prodrug, on satellite cell activity and muscle growth in a piglet model. Satellite cells from tributyrin‐treated piglets had altered myogenic potential, and piglets receiving tributyrin had a ~40% increase in DNA:protein ratio after 21 days, indicating the potential for enhanced muscle growth. To assess muscle growth potential, piglets were supplemented tributyrin (0.5%) during either the neonatal phase (d1–d21) and/or the nursery phase (d21–d58) in a 2 × 2 factorial design. Piglets who received tributyrin during the neonatal phase had improved growth performance at the end of the study and had a ~10% larger loin eye area and muscle fiber cross‐sectional area. Tributyrin treatment in the nursery phase alone did not have a significant effect on muscle growth or feed efficiency. These findings suggest that tributyrin is a potent promoter of muscle growth via altered satellite cell myogenesis.

## Introduction

Postnatal muscle growth and repair rely on the ability of satellite cells, muscle‐resident stem cells, to activate, proliferate, and fuse into growing myofibers (Seale et al. 2000; Dumont et al. 2015). During the neonatal stage of life, the fractional rate of protein synthesis contributing to muscle growth is highest (Davis and Fiorotto 2009), marked by increased satellite cell activation, proliferation (Allbrook et al. 1971; Campion et al. 1981), and myonuclear accretion (Schultz 1996). It has also been found that interventions that target satellite cells at this age can have lifetime effects on muscle growth and regenerative capacity (Alexander et al. 2012; Briggs and Morgan 2013). The myogenic program is under the regulation of a hierarchy of transcription factors that govern satellite cell lineage fate (Rudnicki et al. 2008) and these regulators have proven to be prospective targets in promoting muscle growth (Bentzinger et al. 2012). It has recently been suggested that histone deacetylase (HDAC) inhibitors may serve as a viable tool to epigenetically alter satellite cell behavior (Moresi et al. 2015; Sincennes et al. 2016). Butyrate is a potent and broad‐spectrum inhibitor of HDACs that has shown to be beneficial at treating models of muscle pathology (Minetti et al. 2006; Walsh et al. 2015a), but its effects on satellite cell activity (Leibovitch et al. 1984; Johnston et al. 1992; Iezzi et al. 2002) are controversial. Dietary butyrate, along with tributyrin (a butyrate prodrug), has had positive effects on growth performance (as measured by growth rate and efficiency of growth per unit of feed) (Piva et al. 2002, 2008; Yin et al. 2016), but these findings have been attributed to improved intestinal and digestive functions (Kotunia et al. 2004; Le Gall et al. 2009; He et al. 2015; Huang et al. 2015; Dong et al. 2016). While the favorable use of dietary butyrate seems clear, investigating whether it could be used as a muscle growth promoter would have profound impacts for human health and animal production.

Postnatal muscle growth and protein accretion are considered a hypertrophic event, as the number of muscle fibers is set for most species at the time of birth (Rehfeldt et al. 2000). The activity of satellite cells and their fusion with growing muscle fibers govern the rate of muscle growth and regeneration (Bentzinger et al. 2012). As expected, due to their central role in lifetime muscle growth and regeneration, the activity of satellite cells is tightly regulated via the expression of multiple transcription factors. The paired‐homeobox transcription factor Pax7 is universally expressed in satellite cells and allows for satellite cell proliferation by preventing precocious differentiation (Seale et al. 2000; Oustanina et al. 2004). Additionally, Pax7 plays a role in the regulation of the downstream network of myogenic regulatory factors (MRFs), specifically myogenic differentiation 1 (MyoD) and myogenin (Olguin and Olwin 2004). Changes in the Pax7 and MRF expression patterns have been shown to regulate satellite cell commitment fate, where a decrease in the Pax7:MyoD ratio leads to terminal differentiation and myogenin expression (Olguin et al. 2007). Upregulating Pax7 expression prevents myogenin expression and allows for satellite cells to either self‐renew or enter into a quiescent state (Olguin and Olwin 2004). Activation of MyoD is necessary for proliferating satellite cells to continue through their myogenic lineage and to trigger terminal differentiation of myoblasts (Megeney et al. 1996). The downstream target of MyoD, myogenin, is necessary for fiber development embryonically and for postnatal muscle growth (Hasty et al. 1993; Venuti et al. 1995). Defects in the myogenin gene lead to pools of undifferentiated satellite cells without an apparent effect on the expression of MyoD. The alteration of the expression and timing of these myogenic regulatory genes has been presented as a practical means to increase muscle hypertrophy and regeneration (Blais et al. 2005; Moresi et al. 2015).

Epigenetic modifiers that inhibit HDACs in myoblasts are gaining increasing interest in the fields of muscle growth and regeneration (Sincennes et al. 2016). The beneficial effects of HDAC inhibitors were initially described as a method to treat neoplasms in humans and animal models (see (Marks et al. 2000) for review). Only recently has it been suggested that inhibiting HDACs could alter myogenic programming (McKinsey et al. 2001). When HDACs are expressed in undifferentiated satellite cells, they bind to MyoD and the late‐stage MRF, myogenin, is not expressed (McKinsey et al. 2001). In culture, the HDAC inhibitors have shown to increase myotube hypertrophy without an increase in satellite cell proliferation (Iezzi et al. 2004). Sodium butyrate has also had positive effects at influencing satellite cell fusion and increasing myotube hypertrophy in culture (Iezzi et al. 2002).

The 4‐carbon fatty acid, butyrate, is an inhibitor of many HDACs (Candido et al. 1978; Davie 2003). Butyrate is produced naturally in the body from the fermentation of dietary fiber and has been examined as an antitumor agent since the 1970's (Prasad 1980; Miller et al. 1987). There have been conflicting reports on the effect of in vitro application of butyrate on satellite cell behavior, from enhancing muscle gene expression at different stages of myogenesis (Iezzi et al. 2002) as well as inhibiting some parts of the myogenic program (Fiszman et al. 1980; Leibovitch et al. 1984; Johnston et al. 1992). Within the animal production industry, butyrate has been used as an aid in improving intestinal health (He et al. 2015; Yin et al. 2016) and growth performance through inclusion as the more palatable version, tributyrin, in the diet (Le Gall et al. 2009; Huang et al. 2015). While muscle hypertrophy through satellite cell programming has not been elucidated, butyrate has had promising effects on muscle healing in some injury models (Walsh et al. 2015a,b; Edwards and Butchbach 2016). Using neonatal and nursery piglets as model of rapid muscle growth, we characterized the effects of butyrate on satellite cell activity and their myogenic progression. We hypothesize that supplementation of dietary tributyrin may serve as an effective promoter of muscle growth through enhanced satellite cell myogenesis.

## Materials and Methods

### Animals, diets, and experimental protocol

All animal protocols were approved by the Institutional Animal Care and Use Committee of the University of Maryland‐College Park. In two animal feeding trials, we investigated the effects of tributyrin supplementation on muscle growth during both the neonatal (birth to 21‐days of age) and nursery (22–58‐days of age) phases of growth. During first animal feeding trial, tributyrin was supplemented at two different levels for 21 days to establish an inclusion rate for the second animal feeding trial that would extend into the nursery phase.

#### Study 1

To assess the impact of dietary tributyrin inclusion on in vivo satellite cell programming, 30 cross‐bred female piglets (24 ± 6 h old; 1.79 ± 0.25 kg body weight) were assigned to one of three treatments (*n *= 10/group) and balanced by body weight and litter. Piglets received either a standard commercial milk replacer formula (Advance Liqui‐Wean, Milk Specialties Co., Dundee, IL) where 175 g of dry milk replacer was reconstituted in water to 1 kg total formula (C), or the milk replacer formula supplemented with 0.25% (*T*_0.25_) or 0.5% (*T*_0.5_) total butyric acid in the form of spray‐dried tributyrin (AviPremiumD, Vetagro SpA, Reggio Emilia, Italy). Tributyrin inclusion was on a dry matter basis and diets across treatments were made isoenergetic by the addition coconut oil. Piglets were housed individually and received formula every 2 h (0900–2300) at a limit‐fed rate to match sow reared growth. One piglet from *T*_0.5_ was removed from the study due to nontreatment‐related health issues. Body weight and feed intake were recorded daily for the duration of the 21‐day feeding trial. Piglets were orally administered 20 mg/kg body weight bromodeoxyuiridine (BrdU, Sigma Aldrich, St. Louis, MO) 18 h prior to euthanasia to determine in vivo proliferation of satellite cells. Longissumus dorsi (LD) muscle was used for all tissue analysis and satellite cell isolation.

After the 21‐day neonatal feeding trial, LD was snap frozen in liquid N2 for total DNA, protein abundance, and gene expression analysis. Total muscle protein extractions were performed on ice in NP‐40 buffer (20 mmol/L Tris‐HCl pH 8, 125 mmol/L NaCl, 1% NP‐40, 2 mmol/L ethylenediaminetetra‐acetic acid (EDTA), 10% glycerol) for 30 min containing cOmplete protease inhibitor and PhosSTOP phosphatase inhibitor cocktails (Roche, Basel, Switzerland). Insoluble material was pelleted by centrifugation at 15,000*g* for 15 min at 4°C. Protein concentrations were determined using BCA assays (ThermoFisher Scientific, Waltham, MA).

Total DNA was extracted (DNeasy, Qiagen), fluorescently quantified (Quant‐iT dsDNA assay kit) and compared to the total protein content of the LD muscle. Total RNA was isolated by homogenization using tri‐reagent (ThermoFisher Scientific) with phase separation achieved by chloroform wash. RNA was precipitated with 70% ethanol and transferred to RNeasy spin column and purified according to the manufacturers protocol (Qiagen).

For immunohistological analysis to determine fiber cross‐sectional area (FCA), LD samples were embedded in a 1:1 10% tragacanth gum OCT mixture and snap frozen in liquid N2‐cooled isopentane. Muscles sections (8 *μ*m thick) were cut and mounted on Superfrost Plus slides for immunostaining. FCA and fiber number were determined by anti‐dystrophin staining of the sarcolemma described below.

#### Study 2

We investigated the impact of continual dietary tributyrin inclusion through the nursery phase of growth in a 2 × 2 factorial treatment structure. Piglets, 30 cross‐bred females (24 ± 6 h old; 1.54 ± 0.32 kg body weight), were fed either a standard commercial milk replacer formula (C, *n *= 15) or the milk replacer formula supplemented with 0.5% total butyric acid in the form of spray‐dried tributyrin (T, *n *= 15) (as performed above) for 21 days. Piglets were then weaned at 22‐days of age and crossed into their respective nursery phase dry diet: either commercial dry nursery control diet (CC or TC; *n *= 6/group) (TechMix, Stewart, MN) or a control diet supplemented with 0.5% total butyric acid in the form of spray‐dried tributyrin (CT or TT; *n *= 9/group) (AviPremiumD, Vetagro SpA). The control dry diet was supplemented with microencapsulated palm oil to ensure equivalent energetics to the treatment diet. Piglets were housed individually and fed ad libitum and had free access to ad libitum water. Three piglets (one from the TC group and two from the TT group) were removed from the study for failing to wean. After weaning, body weight and feed intake were recorded weekly for the duration of the 37‐day nursery feeding trial. At the end of the 58‐day feeding trial, LD muscle was removed at the 12th rib to measure total loin area, evaluate gene expression, and for immunohistochemical analysis to determine FCA (as described above).

### Satellite cell isolation and culture

Satellite cells from the neonatal piglets (Study 1) were isolated according to a procedure modified from Doumit and Merkel (1992) and Allen et al. (1997). Briefly, LD muscle was excised from neonatal piglets after their prescribed 21‐day feeding regimen, trimmed of connective tissue, and minced with scissors. Tissue fragments were digested with 1.25 mg/mL protease from *Streptomyces griseous* (Pronase, Sigma‐Aldrich) for 1 h at 37°C. Satellite cells were disassociated from tissue fragments by trituration and differential centrifugation. Cells were preplated on uncoated 15 cm tissue culture dishes for 2 h (37°C, 5% CO_2_) in proliferative growth media (PGM, DMEM + 10% FBS + antibiotics – 100 U/mL penicillin, 100 *μ*g/mL streptomycin, 10 *μ*g/mL gentamycin; Gibco) and then seeded on tissue‐cultured treated dishes coated with Poly‐l‐lysine (100 *μ*g/mL ddH_2_O, Sigma‐Aldrich) and fibronectin (10 *μ*g/mL PBS, Sigma‐Aldrich) in PGM until they reached ~50% confluence (37°C, 5%CO_2_) or analyzed for in vivo proliferation (described below). Cells were then released with 0.05% Trypsin (Gibco) and plated for our studies. Satellite cells were identified by immunostaining for Pax7 (Seale et al. 2000; Zammit et al. 2006); cell isolations >95% Pax7^+^ were used for our studies.

The effect of dietary tributyrin inclusion (0.25% and 0.5%, *n *= 10) effect on ex vivo satellite cell dynamics was analyzed under proliferative and differentiative conditions. Satellite cells were seeded at 2500 cells/cm^2^ in PGM on to plates coated with Poly‐l‐lysine and fibronectin. After a 24‐h attachment period, satellite cells were given 48 h in PGM and then induced to differentiate (DM, DMEM + 2% horse serum; Gibco + antibiotics) for an additional 72 h with complete media changes daily. Total RNA was isolated (RNeasy, Qiagen) at each 24 h time point for gene expression analysis. Satellite cell fusion was measured at 48 h postdifferentiation by immunostaining.

In vitro proliferation was analyzed 24 h after plating using the Click‐iT EdU Alexa Fluor 488 imaging kit (Molecular Probes). Satellite cells were pulsed for 2 h with EdU and then fixed and stained according to the manufacturer's protocol. In vivo proliferation was assayed by identifying proliferating cells with BrdU. Satellite cells that had been direct‐plated were fixed 4% paraformaldehyde and permeablized in 0.1% Triton X‐100 (Sigma‐Aldrich) in PBS. Antigen retrieval was performed with 2 N HCl for 45 m at 37°C, acid was buffered for 10 m with sodium tetraborate, and cells were blocked with 10% goat serum in PBST. Satellite cells were incubated with anti‐BrdU (6 *μ*g mL, Bio‐Techne, Minneapolis, MN) overnight at 4°C. Nuclei were visualized with 4′,6‐diamidino‐2‐phenylindole (DAPI) (Sigma‐Aldrich). Images were visualized on a Zeiss AxioObserver Z.1 and analyzed with ZenPro automated image analysis suite (Carl Zeiss AG, Oberkochen, Germany).

### Western blot analysis

Protein from neonatal LD muscle homogenate was quantified for total protein content by BCA assay (Pierce) and subjected to western blotting. Equal amounts of protein were electrophoresed and separated on 7.5% Mini‐PROTEAN TGX Precast Gels, transferred to an Immun‐Blot PVDF Membrane (Bio‐Rad, Hercules, CA) and stained with SimplyBlue SafeStain (ThermoFisher Scientific) to ensure protein transfer. The membrane was then incubated at 4°C overnight with the following the primary antibodies at a 1:1000 dilution, rabbit anti‐phospho mTOR (Ser‐2448) and rabbit anti‐phospho‐AMPK*α* (Thr‐172) (Cell Signaling Technology, Danvers, MA). Membranes were incubated for 1 h with horseradish peroxidase‐conjugated goat anti‐rabbit secondary antibody (Jackson Immunoresearch, West Grove, PA), and developed with SuperSignal West Pico Chemiluminescent Substrate Kit (ThermoFisher Scientific). Densitometry analysis was performed using a ChemiDoc XRS system and Image Lab Software (BioRad). Equal loading of proteins was confirmed by reprobing with anti‐AMPK*α* and anti‐mTOR antibodies (1:1000, Cell Signaling Technology). Optical density was normalized to a pooled treatment sample as a loading control.

### Analysis of gene expression

Total RNA isolated from neonatal piglet LD muscle and satellite cells were quantified using the Quant‐iT RiboGreen assay (Molecular Probes) according to the manufacturer's protocol. Harvested RNA was reverse transcribed with the SuperScript IV First‐Strand Synthesis System, using equal concentrations of OligodT_(20)_ and random hexamers (Invitrogen) and treated with the RNase H to ensure removal of RNA. The resulting cDNA was quantified with the Quant‐iT OligoGreen assay (Molecular Probes). Total RNA and cDNA quantification were detected on the Synergy HTX microplate reader using the Gen 5.0 v3.0 software (BioTek Instruments, Winooski, VT). cDNA was used for multiplex qRT‐PCR using Bio‐Rad's CFX96 Touch Real‐Time PCR Detection System and iQ Multiplex Powermix. Analysis of gene expression (Pax7, MyoD, myogenin) and amplification plots were executed with the CFX Manager Software (version 3.1, Bio‐Rad). Primers and probes for the gene expression analysis were designed by Integrated DNA Technologies (Coralville, IA) (Table 1). After optimization, a 2:1 primer‐to‐probe ratio was utilized for genes of interest while a 1:1 ratio was used for the reference gene, RPL4. For each assay, samples were amplified for 45 s at 60°C for 40 cycles.

**Table 1:** Primers and probe sequences used for gene expression analysis by multiplex quantitative RT‐PCR

Gene symbol	Gene ID	Primer sequence 5′‐3′	Probe and sequence 5′‐3′
PAX7	100625823	F: CAGCAAGCCCAGACAGG R: TCGGATCTCCCAGCTGAA	(HEX): TTGAGGAGTACAAGAGGGAGAACCCA
MYOD1	407604	F: CCGACGGCATGATGGATTATAG R: CGACACCGCAGCATTCTT	(FAM): AATAGGTGCCGTCGTAGCAGTTCC
MYOG	497618	F: AGTGAATGCAGTTCCCACAG R: AGGTGAGGGAGTGCAGATT	(Texas Red): CAACCCAGGGGATCATCTGCTC
RPL4	100038029	F: TGGTGGTTGAAGATAAAGTTGAAAG R: TGAGAGGCATAAACCTTCTTGAT	(Cy5): AACCAAGGAGGCTGTTCTGCTTCT

### Immunostaining

Satellite cell cultures and LD muscle sections were immunostained to determine myotube formation and FCA, respectively. Satellite cells were analyzed for purity after isolation and for the expression of the contractile protein myosin heavy chain (MyHC) after 48 h of differentiation. Satellite cells were prefixed and nursery LD muscle sections were postfixed in 4% paraformaldehyde and permeablized with Triton X‐100. Samples were blocked with 10% goat serum in PBST (0.1% Tween‐20 in PBS) for 1 h at room temperature. Cells and slides were incubated overnight at 4°C with the primary antibodies mouse monoclonal anti‐Pax7 at 15 *μ*g/mL (Developmental Studies Hybridoma Bank, Iowa City, IA) and mouse monoclonal anti‐MyHC at 10 *μ*g/mL (Roche) or anti‐dystrophin at 5 *μ*g/mL (R&D Systems, Minneapolis, MN), respectively. Primary antibodies were removed and incubated with the secondary antibody (AlexaFluor 488 goat anti‐mouse IgG at 1:500 dilution, Jackson Immunoresearch) in 5% goat serum for 1 h at room temperature. Myotube formation and FCA images were collected with Zeiss AxioObserver Z.1 and analyzed with ZenPro automated image analysis suite (Carl Zeiss AG).

### Statistical analysis

#### Study 1

The effects of tributyrin concentration on the response variables were analyzed using an *F*‐test in ANOVA (GraphPad Prism 7, GraphPad Software, Inc., La Jolla, CA).

#### Study 2

Data were analyzed as a two‐way ANOVA using the PROC MIXED procedure in SAS (version 9.3; SAS Institute Inc., Cary, NC). Milk replacer treatment, dry nursery dietary treatment, and their interaction were analyzed as fixed effects while covariate(s) (initial body weight and/or weaning weight) were analyzed as random effects in the MIXED procedure of SAS (9.3).

In the case of a significant F‐test, multiple mean comparisons were analyzed using a Tukey's adjustment. A probability of *P *≤ 0.05 was considered significant and a *P*‐value between 0.05 and 0.10 (0.05 < *P *≤ 0.10) was considered a trend. Data reported as least square means ± SEM.

## Results

### Tributyrin inclusion on growth performance

#### Study 1

**Figure 1:**
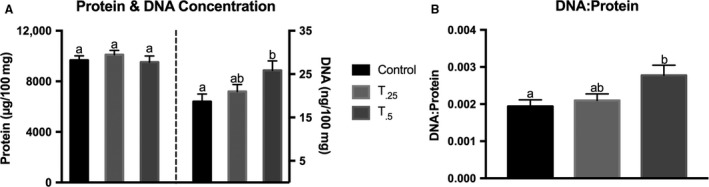
Total protein and DNA were extracted from *Longissimus dorsi* muscle of piglets fed either a basal milk replacer (*n *= 10), or the milk replacer supplemented with 0.25% (*T*_0.25_, *n *= 10) or 0.5% (*T*_0.5_, *n *= 9) tributyrin for 21 days. (A) Protein and DNA concentrations in LD muscle tissue. (B) Bar graph depicting the ratio of DNA:Protein. Bars not sharing a common superscript differ significantly, *P *< 0.05.

After the 21‐day neonatal feeding trial, there was no effect of treatment on final body weight, average daily gain (ADG), or feed efficiency. There was a nonsignificant decrease in feed intake in the *T*_0.5_ group. LD muscle was harvested to analyze treatment effects on cellular mechanisms of muscle growth rate. Tissue homogenate was examined for total protein and DNA content to assess the DNA:protein as a measure of myonuclear accretion (Fig. 1). There was a significant increase in the DNA:protein in the *T*_0.5_ group (5.5 ± 0.5 mg/g, DNA/protein) compared to the other two treatment diets (*T*_0.25_ = 4.1 ± 0.4 mg/g; C = 3.8 ± 0.5 mg/g) (*P* < 0.05). There was no treatment effect on the ratio of phosphorylated to total mTOR or AMPK*α* protein expression revealed by western blotting (data not shown). Based on these findings, we supplemented the milk replacer with 0.5% tributyrin for the nursery feeding trial in order to investigate the potential for enhanced muscle growth.

#### Study 2

**Figure 2:**
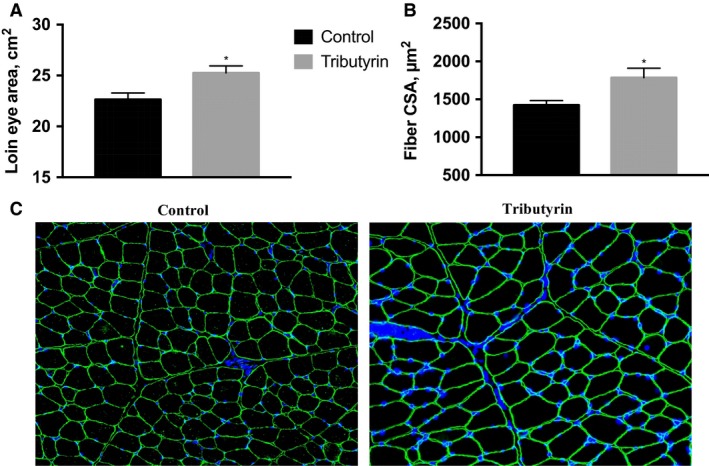
At 58 days of age, cross‐section of *Longissimus dorsi* (LD) muscle was taken at the 12th rib and utilized for immunohistochemical analysis to determine fiber cross‐sectional area (FCA). Values depicted are based off pooled neonatal control (C, *n *= 12) or tributyrin (T, *n *= 12) treatment groups. (A) The cross‐sectional area of the LD at the 12th rib (loin eye). (B) LD muscle FCA as determined by immunohistochemistry. (C) Immunohistochemical analysis of FCA of the LD muscle. Muscle fibers were cryosectioned and stained with anti‐dystrophin to visualize sarcolemma (green), >400 fibers/animal were counted using Zeiss ZEN Pro automated image analysis. Nuclei were visualized with DAPI. Significance was declared at *P *< 0.05 (*).

At the end of the 58‐day feeding trial (neonatal + nursery), there was a significant increase in final body weight and ADG in animals that received tributyrin in the milk replacer before weaning (TT and TC) compared to animals that received control milk replacer diet (CC and CT) (*P *< 0.05; Table 2). After completion of the trial, piglets treated with tributyrin during the neonatal phase (D1–D21) weighed 8% more than the control piglets, 30.8 ± 0.6 kg and 28.4 ± 0.6 kg, respectively, and had a 9% increase in their ADG (659 ± 17 g compared to the control 603 ± 16 g). There were no treatment effects seen in final body weight or feed efficiency between the four nursery diet treatment comparisons. Loin area and FCA from the piglets treated with tributyrin during the neonatal phase was significantly larger compared to those piglets that did not receive tributyrin in their milk replacer (*P* < 0.05; Fig. 2A). At the end of the 58‐day feeding trial, piglets supplemented with tributyrin during the neonatal period had a loin area of 25.3 ± 0.7 cm^2^ compared to the control piglets, 22.7 ± 0.6 cm^2^. Similarly, muscle histology sections stained with anti‐dystrophin (Fig. 2C) revealed a 25% increase in the FCA of TT and TC piglets (1790 ± 120 *μ*m^2^) compared to CC and CT piglets (1420 ± 60 *μ*m^2^) (*P* < 0.05; Fig. 2B).

**Table 2:** Effects of tributyrin supplementation during neonatal and nursery phases on growth performance

	Treatments^a^^,^ b				SEM	*P* d		
	CC	CT	TC	TT		Milk	Dry	Milk × dry^a^
*n*	6	9	5	7				
Initial body weight (kg)	1.6	1.5	1.4	1.6	0.06	–	–	–
Weaning weight (kg)	6.4	6.3	5.9	6	0.1	0.11	–	–
Final body weight (kg)	29.6	29.3	30.4	29.6	1.1	**0.03**	0.66	1.00
Postweaning average daily gain (g)	627	618	658	641	28	**0.03**	0.66	0.98
Feed efficiency after weaning^c^	1.41	1.41	1.37	1.41	0.04	0.37	0.52	0.99

a Value represented as least square means of main effects.

b CC (milk replacer control; nursery control), CT (milk replacer control; nursery diet with tributyrin), TC (milk replacer with tributyrin; nursery control diet), and TT (milk replacer with tributyrin; nursery diet with tributyrin).

c Feed efficiency = Average daily feed intake/average daily gain (day 22–58).

d Effect of tributyrin inclusion in the milk replacer, in the nursery diet, and their interaction.

Bold values indicate the level of significance.

### Satellite cell myogenesis

**Figure 3:**
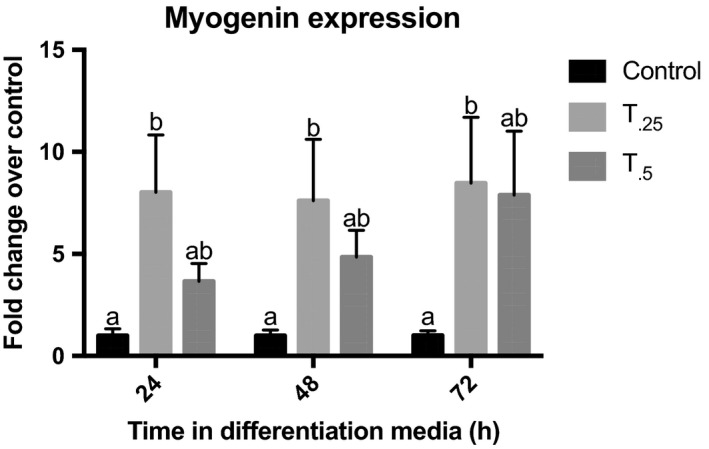
Myogenin gene expression in cultured satellite cells from neonatal piglets treated with either control diet (*n *= 10), or a control diet supplemented with 0.25% (*T*_0.25_, *n *= 10) or 0.5% (*T*_0.5_, *n *= 9) tributyrin for 21 days. After induced to differentiate, total RNA was harvested and myogenin expression was quantified by RT‐PCR. Expression was normalized within animal to RPL4 at each time point. Bars not sharing superscripts differ significantly, *P *< 0.05.

**Figure 4:**
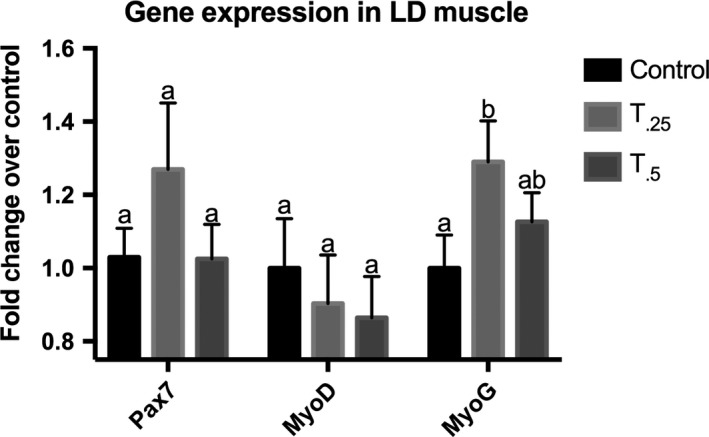
Total RNA was harvested from *Longissiumus dorsi* muscle from neonatal piglets treated with either control diet (*n *= 10), or a control diet supplemented with 0.25% (*T*_0.25_, *n *= 10) or 0.5% (*T*_0.5_, *n *= 9) tributyrin for 21 days. Gene expression of Pax7 and the myogenic regulatory factors MyoD and myogenin (MyoG) was measured by quantitative RT‐PCR. Expression was normalized within animal to RPL4. Bars not sharing superscripts showed a trend, *P *< 0.10.

**Figure 5:**
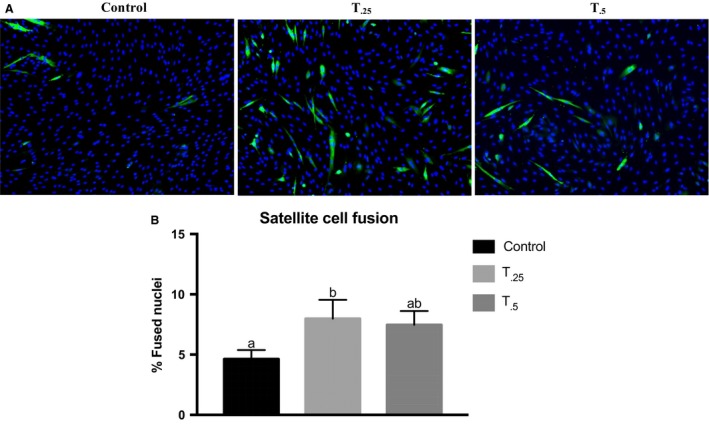
Satellite cells harvested from neonatal piglets treated with a control milk replacer diet (*n *= 10), or a control diet supplemented with 0.25% (*T*_0.25_, *n *= 10) or 0.5% (*T*_0.5_, *n *= 9) tributyrin for 21 days were induced to differentiate for 48 h. (A) Representative images of immunofluorescently stained myotubes with anti‐MyHC (green). Nuclei were visualized with DAPI and counted using Zeiss ZEN Pro automated image analysis. (B) Bar graph depicting the fusion percentages (≥2 nuclei expressing MyHC/total nuclei) in satellite cells from those animals treated with either control diet, or a control diet supplemented with 0.25% (*T*_0.25_) or 0.5% (*T*_0.5_) tributyrin. Bars not sharing superscripts differ significantly, *P *< 0.05.

Satellite cells were harvested from neonatal piglets after 21 days of milk feeding with (*T*_0.25_ or *T*_0.5_) or without (C) tributyrin supplementation. There was no treatment effect on in vivo or in vitro satellite cell proliferation, as assessed by BrdU and EdU staining, respectively. Satellite cells were cultured under proliferative conditions until confluent and induced to differentiate with gene expression analyzed every 24 h to determine myogenic progression. Throughout differentiation, there was an average sevenfold (*T*_0.25_; *P < *0.01) and fourfold (*T*_0.5_; *P* *< *0.05) increase in the expression of the late‐stage MRF myogenin in satellite cells from those animals treated with tributyrin compared to control animals (Fig. 3). There was not a significant change in Pax7 or MyoD expression in satellite cells from treated animals compared to the control animals (data not shown). When LD muscle sections of neonatal piglets from study 1 were analyzed for myogenic gene expression, a similar trend was revealed. Myogenin was upregulated 1.4‐fold in the *T*_0.25_ group compared to the control (*P* < 0.10) without changes in Pax7 or MyoD expression (Fig 4). To further examine the effect of tributyrin on ex vivo satellite cell myogenesis, myotube formation was determined 48 h after satellite cells were induced to differentiate (Fig. 5A). There was a 1.7‐fold increase in the number of MyHC^+^‐fused nuclei from *T*_0.25_ animals compared with the control animals (*P *< 0.05; Fig. 5B).

## Discussion

The benefits of dietary inclusion of butyrate or tributyrin on animal health (Galfi and Bokori 1990; Piva et al. 2002, 2008; Hou et al. 2014) and growth performance (Leeson et al. 2005; Lu et al. 2012; Piva et al. 2016; Bedford et al. 2017) have been demonstrated; however, the role that butyrate has on the mechanisms behind muscle growth has yet to be elucidated. In the present study, we examined the ability of dietary tributyrin to enhance muscle hypertrophy and its effect on satellite cell programming. Our results indicate that early‐life supplementation with tributyrin may promote muscle growth through increased satellite cell myogenic potential.

The objective of study 1 was to assess what dietary tributyrin inclusion level is necessary to impact muscle growth parameters. Also, satellite cells were harvested from the neonatal animals and we investigated the effect of tributyrin supplementation on in vivo and ex vivo satellite cell programming. At the completion of study 1, the results of tributyrin inclusion during neonatal feeding were suggestive that an inclusion rate of tributyrin at 0.5% in study 2 may improve muscle growth through enhanced myonuclear accretion.

Studies have revealed the beneficial performance and growth effects of butyrate and tributyrin supplementation, all the while linking these positive results to an enhanced trophic status of the GI tract. Tributyrin provides an efficient means to deliver butyrate to systemic circulation for a sustained duration (Egorin et al. 1999). Our results indicate that butyrate may act as a molecular signal, with a direct effect on satellite cell myogenesis. Our primary objective was to determine the effect of tributyrin supplementation on muscle growth at different stages of early life (preweaning/postweaning). Postnatal muscle growth and regeneration are dependent upon satellite cell activation and proliferation followed by consequent differentiation and fusion into the growing myofiber (Davis and Fiorotto 2009). After being fed a milk replacer supplemented with tributyrin for 21 days, piglets had an increase in total DNA content and the DNA:protein. While there were no differences in weight gain at the end of the neonatal feeding trial, an increase of almost 40% in total DNA content and the DNA:protein ratio suggests an amplification of myonuclear accretion and a propensity for enhanced muscle growth (Brown and Stickland 1994). Secondary to myonuclear accretion is the subsequent protein synthesis and muscle fiber hypertrophy. During the neonatal feeding trial, piglets were limit‐fed to match normal, sow reared growth. Given this feeding regimen, it is possible that the piglets were limited in amino acid availability for maximal protein synthesis. While it seems that tributyrin supplementation altered satellite cell behavior, there did not appear to be any changes in the mTOR signaling pathway, which governs protein synthesis (Bodine et al. 2001) and has been shown to regulate satellite cell fusion machinery (Sun et al. 2010). In intestinal epithelial cells, butyrate has been shown to reduce the amount of phospho‐AMPK which is known to inhibit mTOR (Yan and Ajuwon 2017); however, we did not see any differences by tributyrin treatment. The time required for the accumulated myonuclei to synthesize protein and contribute to the growing fiber may not have been sufficient to realize increased muscle hypertrophy. These results imply that prolonged, postweaning ad libitum feeding in the nursery would be necessary to assess whether dietary tributyrin inclusion would effectively promote muscle growth.

Neonatal piglets were once again fed a milk replacer supplemented with 0.5% tributyrin and then weaned into the nursery on a dry diet supplemented with 0.5% tributyrin. After the nursery feeding trial, animals that had received tributyrin supplementation in their milk had a significant increase in ADG and final body weight. Interestingly, there was not a treatment effect with regards to nursery diet. These results are similar to those obtained by Le Gall et al. (2009), where those animals that had received butyrate during milk feeding had enhanced growth, but the authors again attribute the increase in growth to increased feed digestibility and increased feed intake. Our data showed no significant differences in feed efficiency; however, animals receiving tributyrin had a reduction in feed consumed, but the differences did not rise to the level of significance. Butyrate supplementation has been associated with decreased feed intake while on treatment diets in other studies (Leeson et al. 2005; Hou et al. 2014); there is also a body of evidence linking mild ketosis with decreased appetite and increased satiety through unknown mechanisms (Puchalska and Crawford 2017). It may be that exogenous supplementation of butyrate induces a mild ketosis of which decreases overall feed intake and may confound the beneficial effects of tributyrin supplementation preweaning. However, the positive effects of tributyrin supplementation in the piglet milk replacer resulting in an increase in growth performance (Final BW = 8%, ADG = 9%) and muscle growth (Loin area = 11%, FCA = 25%) were unmistakable after piglets had reached the end of the nursery feeding.

We found that the hypertrophic benefit of tributyrin supplementation altered satellite cell behavior and enhanced terminal differentiation. The increase in myogenin expression found in the LD muscle of the tributyrin‐treated neonatal piglets may be indicative of satellite cells beginning to differentiate quicker and fuse into present myofibers (Wang and Rudnicki 2012). This was marked by the enhanced terminal differentiation seen in the ex vivo satellite cell cultures. Satellite cells from those piglets treated with tributyrin (both *T*_0.25_ and *T*_0.5_) had an increase in the proportion of those cells expression of the contractile protein MyHC. This was associated with the increased upregulation of myogenin after satellite cell cultures were induced to differentiate. Although there were no noticeable treatment effects on either in vivo or ex vivo satellite cell proliferation, this is noteworthy due to butyrate's ability to halt cell proliferation seen in satellite cell culture experiments (Leibovitch et al. 1984; Iezzi et al. 2002). These results indicate that the increased myonuclear content may not come from a significant increase in the proliferative potential of satellite cells, but rather a temporal acceleration of the differentiation process. The lack of differences seen in the in vivo proliferation of satellite cells also suggests that tributyrin's effect on satellite cell behavior may be most salient at the earliest stages of life.

The differences in gene expression and myogenic potential displayed in the satellite cell cultures from the tributyrin‐treated groups also support the notion that tributyrin, and its ensuing metabolite butyrate, may be serving as an epigenetic modifier of satellite cell behavior (Sincennes et al. 2016). The HDAC inhibitory properties of butyrate may be leading to improved muscle differentiation through histone modifications that result in increased myogenin expression. This compliments the findings that decreased HDAC activity has resulted in increased acetylation of nonhistone proteins, such as MyoD, which is necessary for myogenesis and promotes myogenic differentiation (Mal et al. 2001; Ma et al. 2005; Duquet et al. 2006). In this regard, it appears that dietary tributyrin may serve as a viable inhibitor of HDACs for pharmacological manipulation of myogenic genes.

Using a neonatal piglet model of muscle growth, we have shown that early dietary inclusion of the butyrate prodrug, tributyrin, resulted in an increase in muscle mass by muscle fiber hypertrophy. Our findings also indicate that the accelerated muscle growth triggered by tributyrin is due to increased myonuclear accretion and subsequent myofiber hypertrophy. Supplementing tributyrin in the milk replacer formula of neonatal piglets resulted in enhanced muscle growth driven by enhanced satellite cell myogenesis. Contradictory with some previous findings (Le Gall et al. 2009; Piva et al. 2016), dietary supplementation of tributyrin to the older weaned pigs did not increase muscle growth or improve growth performance. This suggests that there is a window of opportunity to utilize tributyrin to impact muscle growth via alterations in satellite cell activity and that early‐life interventions with tributyrin may be able to ameliorate deficits in muscle growth caused by limitations in the myogenic activity of satellite cells.

## Conflict of Interest

No conflicts of interests, financial or otherwise, are declared by the authors.

## References

[phy213706-bib-0001] Alexander, L. S. , B. S. Seabolt , R. P. Rhoads , and C. H. Stahl . 2012 Neonatal phosphate nutrition alters in vivo and in vitro satellite cell activity in pigs. Nutrients 4:436–448. 22822445 10.3390/nu4060436 PMC3397345

[phy213706-bib-0002] Allbrook, D. B. , M. F. Han , and A. E. Hellmuth . 1971 Population of muscle satellite cells in relation to age and mitotic activity. Pathology 3:223–243. 10.3109/00313027109073739 4107201

[phy213706-bib-0003] Allen, R. E. , C. J. Temm‐Grove , S. M. Sheehan , and G. Rice . 1997 Skeletal muscle satellite cell cultures. Methods Cell Biol. 52:155–176. 9379949 10.1016/s0091-679x(08)60378-7

[phy213706-bib-0004] Bedford, A. , H. Yu , E. J. Squires , S. Leeson , and J. Gong . 2017 Effects of supplementation level and feeding schedule of butyrate glycerides on the growth performance and carcass composition of broiler chickens. Poult. Sci. 96:3221–3228. 28431158 10.3382/ps/pex098

[phy213706-bib-0005] Bentzinger, C. F. , Y. X. Wang , and M. A. Rudnicki . 2012 Building muscle: molecular regulation of myogenesis. Cold Spring Harb. Perspect. Biol. 4:p. a008342. 10.1101/cshperspect.a008342 PMC3281568 22300977

[phy213706-bib-0006] Blais, A. , M. Tsikitis , D. Acosta‐Alvear , R. Sharan , Y. Kluger , and B. D. Dynlacht . 2005 An initial blueprint for myogenic differentiation. Genes Dev. 19:553–569. 15706034 10.1101/gad.1281105 PMC551576

[phy213706-bib-0007] Bodine, S. C. , T. N. Stitt , M. Gonzalez , W. O. Kline , G. L. Stover , R. Bauerlein , et al. 2001 Akt/mTOR pathway is a crucial regulator of skeletal muscle hypertrophy and can prevent muscle atrophy in vivo. Nat. Cell Biol. 3:1014–1019. 11715023 10.1038/ncb1101-1014

[phy213706-bib-0008] Briggs, D. , and J. E. Morgan . 2013 Recent progress in satellite cell/myoblast engraftment – relevance for therapy. FEBS J. 280:4281–4293. 23560812 10.1111/febs.12273 PMC3795440

[phy213706-bib-0009] Brown, S. C. , and N. C. Stickland . 1994 Muscle at birth in mice selected for large and small body size. J. Anat. 184(Pt 2):371–380. 8014128 PMC1259997

[phy213706-bib-0010] Campion, D. R. , R. L. Richardson , J. O. Reagan , and R. R. Kraeling . 1981 Changes in the satellite cell population during postnatal growth of pig skeletal muscle. J. Anim. Sci. 52:1014–1018. 7240043 10.2527/jas1981.5251014x

[phy213706-bib-0011] Candido, E. P. , R. Reeves , and J. R. Davie . 1978 Sodium butyrate inhibits histone deacetylation in cultured cells. Cell 14:105–113. 667927 10.1016/0092-8674(78)90305-7

[phy213706-bib-0012] Davie, J. R. 2003 Inhibition of histone deacetylase activity by butyrate. J. Nutr. 133:2485S–2493S. 12840228 10.1093/jn/133.7.2485S

[phy213706-bib-0013] Davis, T. A. , and M. L. Fiorotto . 2009 Regulation of muscle growth in neonates. Curr. Opin. Clin. Nutr. Metab. Care 12:78–85. 19057192 10.1097/MCO.0b013e32831cef9f PMC2653196

[phy213706-bib-0014] Dong, L. , X. Zhong , J. He , L. Zhang , K. Bai , W. Xu , et al. 2016 Supplementation of tributyrin improves the growth and intestinal digestive and barrier functions in intrauterine growth‐restricted piglets. Clin. Nutr. 35:399–407. 26112894 10.1016/j.clnu.2015.03.002

[phy213706-bib-0015] Doumit, M. E. , and R. A. Merkel . 1992 Conditions for isolation and culture of porcine myogenic satellite cells. Tissue Cell 24:253–262. 1589873 10.1016/0040-8166(92)90098-r

[phy213706-bib-0016] Dumont, N. A. , C. F. Bentzinger , M. C. Sincennes , and M. A. Rudnicki . 2015 Satellite cells and skeletal muscle regeneration. Compr. Physiol. 5:1027–1059. 26140708 10.1002/cphy.c140068

[phy213706-bib-0017] Duquet, A. , A. Polesskaya , S. Cuvellier , S. Ait‐Si‐Ali , P. Hery , L. L. Pritchard , et al. 2006 Acetylation is important for MyoD function in adult mice. EMBO Rep. 7:1140–1146. 17028574 10.1038/sj.embor.7400820 PMC1679786

[phy213706-bib-0018] Edwards, J. D. , and M. E. Butchbach . 2016 Effect of the butyrate prodrug pivaloyloxymethyl butyrate (AN9) on a mouse model for spinal muscular atrophy. J. Neuromuscul. Dis. 3:511–515. 27911337 10.3233/JND-160187 PMC5184770

[phy213706-bib-0019] Egorin, M. J. , Z. M. Yuan , D. L. Sentz , K. Plaisance , and J. L. Eiseman . 1999 Plasma pharmacokinetics of butyrate after intravenous administration of sodium butyrate or oral administration of tributyrin or sodium butyrate to mice and rats. Cancer Chemother. Pharmacol. 43:445–453. 10321503 10.1007/s002800050922

[phy213706-bib-0020] Fiszman, M. Y. , D. Montarras , W. Wright , and F. Gros . 1980 Expression of myogenic differentiation and myotube formation by chick embryo myoblasts in the presence of sodium butyrate. Exp. Cell Res. 126:31–37. 7358093 10.1016/0014-4827(80)90467-x

[phy213706-bib-0021] Galfi, P. , and J. Bokori . 1990 Feeding trial in pigs with a diet containing sodium n‐butyrate. Acta Vet. Hung. 38:3–17. 2100936

[phy213706-bib-0022] Hasty, P. , A. Bradley , J. H. Morris , D. G. Edmondson , J. M. Venuti , E. N. Olson , et al. 1993 Muscle deficiency and neonatal death in mice with a targeted mutation in the myogenin gene. Nature 364:501–506. 8393145 10.1038/364501a0

[phy213706-bib-0023] He, J. , L. Dong , W. Xu , K. Bai , C. Lu , Y. Wu , et al. 2015 Dietary tributyrin supplementation attenuates insulin resistance and abnormal lipid metabolism in suckling piglets with intrauterine growth retardation. PLoS ONE 10:e0136848. 26317832 10.1371/journal.pone.0136848 PMC4552672

[phy213706-bib-0024] Hou, Y. , L. Wang , D. Yi , B. Ding , X. Chen , Q. Wang , et al. 2014 Dietary supplementation with tributyrin alleviates intestinal injury in piglets challenged with intrarectal administration of acetic acid. Br. J. Nutr. 111:1748–1758. 24506942 10.1017/S0007114514000038

[phy213706-bib-0025] Huang, C. , P. Song , P. Fan , C. Hou , P. Thacker , and X. Ma . 2015 Dietary sodium butyrate decreases postweaning diarrhea by modulating intestinal permeability and changing the bacterial communities in weaned piglets. J. Nutr. 145:2774–2780. 26491121 10.3945/jn.115.217406

[phy213706-bib-0026] Iezzi, S. , G. Cossu , C. Nervi , V. Sartorelli , and P. L. Puri . 2002 Stage‐specific modulation of skeletal myogenesis by inhibitors of nuclear deacetylases. Proc. Natl Acad. Sci. USA 99:7757–7762. 12032356 10.1073/pnas.112218599 PMC124343

[phy213706-bib-0027] Iezzi, S. , M. Di Padova , C. Serra , G. Caretti , C. Simone , E. Maklan , et al. 2004 Deacetylase inhibitors increase muscle cell size by promoting myoblast recruitment and fusion through induction of follistatin. Dev. Cell 6:673–684. 15130492 10.1016/s1534-5807(04)00107-8

[phy213706-bib-0028] Johnston, L. A. , S. J. Tapscott , and H. Eisen . 1992 Sodium butyrate inhibits myogenesis by interfering with the transcriptional activation function of MyoD and myogenin. Mol. Cell. Biol. 12:5123–5130. 1328872 10.1128/mcb.12.11.5123-5130.1992 PMC360446

[phy213706-bib-0029] Kotunia, A. , J. Wolinski , D. Laubitz , M. Jurkowska , V. Rome , P. Guilloteau , et al. 2004 Effect of sodium butyrate on the small intestine development in neonatal piglets fed [correction of feed] by artificial sow. J. Physiol. Pharmacol. 55(Suppl 2):59–68. 15608361

[phy213706-bib-0030] Le Gall, M. , M. Gallois , B. Seve , I. Louveau , J. J. Holst , I. P. Oswald , et al. 2009 Comparative effect of orally administered sodium butyrate before or after weaning on growth and several indices of gastrointestinal biology of piglets. Br. J. Nutr. 102:1285–1296. 19480733 10.1017/S0007114509990213

[phy213706-bib-0031] Leeson, S. , H. Namkung , M. Antongiovanni , and E. H. Lee . 2005 Effect of butyric acid on the performance and carcass yield of broiler chickens. Poult. Sci. 84:1418–1422. 16206563 10.1093/ps/84.9.1418

[phy213706-bib-0032] Leibovitch, M. P. , S. A. Leibovitch , M. Raymondjean , and J. Kruh . 1984 Effect of sodium butyrate on gene expression in a rat myogenic cell line. Biochem. Biophys. Res. Commun. 125:1129–1136. 6517941 10.1016/0006-291x(84)91401-3

[phy213706-bib-0033] Lu, H. , S. Su , and K. M. Ajuwon . 2012 Butyrate supplementation to gestating sows and piglets induces muscle and adipose tissue oxidative genes and improves growth performance. J. Anim. Sci. 90(Suppl 4):430–432. 23365400 10.2527/jas.53817

[phy213706-bib-0034] Ma, K. , J. K. Chan , G. Zhu , and Z. Wu . 2005 Myocyte enhancer factor 2 acetylation by p300 enhances its DNA binding activity, transcriptional activity, and myogenic differentiation. Mol. Cell. Biol. 25:3575–3582. 15831463 10.1128/MCB.25.9.3575-3582.2005 PMC1084296

[phy213706-bib-0035] Mal, A. , M. Sturniolo , R. L. Schiltz , M. K. Ghosh , and M. L. Harter . 2001 A role for histone deacetylase HDAC1 in modulating the transcriptional activity of MyoD: inhibition of the myogenic program. EMBO J. 20:1739–1753. 11285237 10.1093/emboj/20.7.1739 PMC145490

[phy213706-bib-0036] Marks, P. A. , V. M. Richon , and R. A. Rifkind . 2000 Histone deacetylase inhibitors: inducers of differentiation or apoptosis of transformed cells. J. Natl Cancer Inst. 92:1210–1216. 10922406 10.1093/jnci/92.15.1210

[phy213706-bib-0037] McKinsey, T. A. , C. L. Zhang , and E. N. Olson . 2001 Control of muscle development by dueling HATs and HDACs. Curr. Opin. Genet. Dev. 11:497–504. 11532390 10.1016/s0959-437x(00)00224-0

[phy213706-bib-0038] Megeney, L. A. , B. Kablar , K. Garrett , J. E. Anderson , and M. A. Rudnicki . 1996 MyoD is required for myogenic stem cell function in adult skeletal muscle. Genes Dev. 10:1173–1183. 8675005 10.1101/gad.10.10.1173

[phy213706-bib-0039] Miller, A. A. , E. Kurschel , R. Osieka , and C. G. Schmidt . 1987 Clinical pharmacology of sodium butyrate in patients with acute leukemia. Eur. J. Cancer Clin. Oncol. 23:1283–1287. 3678322 10.1016/0277-5379(87)90109-x

[phy213706-bib-0040] Minetti, G. C. , C. Colussi , R. Adami , C. Serra , C. Mozzetta , V. Parente , et al. 2006 Functional and morphological recovery of dystrophic muscles in mice treated with deacetylase inhibitors. Nat. Med. 12:1147–1150. 16980968 10.1038/nm1479

[phy213706-bib-0041] Moresi, V. , N. Marroncelli , and S. Adamo . 2015 New insights into the epigenetic control of satellite cells. World J. Stem Cells 7:945–955. 26240681 10.4252/wjsc.v7.i6.945 PMC4515437

[phy213706-bib-0042] Olguin, H. C. , and B. B. Olwin . 2004 Pax‐7 up‐regulation inhibits myogenesis and cell cycle progression in satellite cells: a potential mechanism for self‐renewal. Dev. Biol. 275:375–388. 15501225 10.1016/j.ydbio.2004.08.015 PMC3322464

[phy213706-bib-0043] Olguin, H. C. , Z. Yang , S. J. Tapscott , and B. B. Olwin . 2007 Reciprocal inhibition between Pax7 and muscle regulatory factors modulates myogenic cell fate determination. J. Cell Biol. 177:769–779. 17548510 10.1083/jcb.200608122 PMC2064278

[phy213706-bib-0044] Oustanina, S. , G. Hause , and T. Braun . 2004 Pax7 directs postnatal renewal and propagation of myogenic satellite cells but not their specification. EMBO J. 23:3430–3439. 15282552 10.1038/sj.emboj.7600346 PMC514519

[phy213706-bib-0045] Piva, A. , A. Prandini , L. Fiorentini , M. Morlacchini , F. Galvano , and J. B. Luchansky . 2002 Tributyrin and lactitol synergistically enhanced the trophic status of the intestinal mucosa and reduced histamine levels in the gut of nursery pigs. J. Anim. Sci. 80:670–680. 11890403 10.2527/2002.803670x

[phy213706-bib-0046] Piva, A. , E. Grilli , L. Fabbri , V. Pizzamiglio , P. P. Gatta , F. Galvano , et al. 2008 Intestinal metabolism of weaned piglets fed a typical United States or European diet with or without supplementation of tributyrin and lactitol. J. Anim. Sci. 86:2952–2961. 18502885 10.2527/jas.2007-0402

[phy213706-bib-0047] Piva, A. , M. Morlacchini , G. Casadei , P. P. Gatta , G. Biagi , and A. Prandini . 2016 Sodium butyrate improves growth performance of weaned piglets during the first period after weaning. Ital. J. Anim. Sci. 1:35–41.

[phy213706-bib-0048] Prasad, K. N. 1980 Butyric acid: a small fatty acid with diverse biological functions. Life Sci. 27:1351–1358. 7003281 10.1016/0024-3205(80)90397-5

[phy213706-bib-0049] Puchalska, P. , and P. A. Crawford . 2017 Multi‐dimensional roles of ketone bodies in fuel metabolism, signaling, and therapeutics. Cell Metab. 25:262–284. 28178565 10.1016/j.cmet.2016.12.022 PMC5313038

[phy213706-bib-0050] Rehfeldt, C. , I. Fiedler , G. Dietl , and K. Ender . 2000 Myogenesis and postnatal skeletal muscle cell growth as influenced by selection. Livest. Prod. Sci. 66:177–188.

[phy213706-bib-0051] Rudnicki, M. A. , F. Le Grand , I. McKinnell , and S. Kuang . 2008 The molecular regulation of muscle stem cell function. Cold Spring Harb. Symp. Quant. Biol. 73:323–331. 19329572 10.1101/sqb.2008.73.064

[phy213706-bib-0052] Schultz, E. 1996 Satellite cell proliferative compartments in growing skeletal muscles. Dev. Biol. 175:84–94. 8608871 10.1006/dbio.1996.0097

[phy213706-bib-0053] Seale, P. , L. A. Sabourin , A. Girgis‐Gabardo , A. Mansouri , P. Gruss , and M. A. Rudnicki . 2000 Pax7 is required for the specification of myogenic satellite cells. Cell 102:777–786. 11030621 10.1016/s0092-8674(00)00066-0

[phy213706-bib-0054] Sincennes, M. C. , C. E. Brun , and M. A. Rudnicki . 2016 Concise review: epigenetic regulation of myogenesis in health and disease. Stem Cells Transl. Med. 5:282–290. 26798058 10.5966/sctm.2015-0266 PMC4807671

[phy213706-bib-0055] Sun, Y. , Y. Ge , J. Drnevich , Y. Zhao , M. Band , and J. Chen . 2010 Mammalian target of rapamycin regulates miRNA‐1 and follistatin in skeletal myogenesis. J. Cell Biol. 189:1157–1169. 20566686 10.1083/jcb.200912093 PMC2894448

[phy213706-bib-0056] Venuti, J. M. , J. H. Morris , J. L. Vivian , E. N. Olson , and W. H. Klein . 1995 Myogenin is required for late but not early aspects of myogenesis during mouse development. J. Cell Biol. 128:563–576. 7532173 10.1083/jcb.128.4.563 PMC2199898

[phy213706-bib-0057] Walsh, M. E. , A. Bhattacharya , Y. Liu , and H. Van Remmen . 2015a Butyrate prevents muscle atrophy after sciatic nerve crush. Muscle Nerve 52:859–868. 25727783 10.1002/mus.24622

[phy213706-bib-0058] Walsh, M. E. , A. Bhattacharya , K. Sataranatarajan , R. Qaisar , L. Sloane , M. M. Rahman , et al. 2015b The histone deacetylase inhibitor butyrate improves metabolism and reduces muscle atrophy during aging. Aging Cell 14:957–970. 26290460 10.1111/acel.12387 PMC4693467

[phy213706-bib-0059] Wang, Y. X. , and M. A. Rudnicki . 2012 Satellite cells, the engines of muscle repair. Nat. Rev. Mol. Cell Biol. 13:127–133. 10.1038/nrm3265 22186952

[phy213706-bib-0060] Yan, H. , and K. M. Ajuwon . 2017 Butyrate modifies intestinal barrier function in IPEC‐J2 cells through a selective upregulation of tight junction proteins and activation of the Akt signaling pathway. PLoS ONE 12:e0179586. 28654658 10.1371/journal.pone.0179586 PMC5487041

[phy213706-bib-0061] Yin, F. , H. Yu , D. Lepp , X. Shi , X. Yang , J. Hu , et al. 2016 Transcriptome analysis reveals regulation of gene expression for lipid catabolism in young broilers by butyrate glycerides. PLoS ONE 11:e0160751. 27508934 10.1371/journal.pone.0160751 PMC4979964

[phy213706-bib-0062] Zammit, P. S. , F. Relaix , Y. Nagata , A. P. Ruiz , C. A. Collins , T. A. Partridge , et al. 2006 Pax7 and myogenic progression in skeletal muscle satellite cells. J. Cell Sci. 119:1824–1832. 16608873 10.1242/jcs.02908

